# Suspected Emphysematous Cellulitis of the Face Secondary to Untreated Nondisplaced Angle of the Mandible Fracture: A Case Report

**DOI:** 10.1155/crid/6247721

**Published:** 2025-04-21

**Authors:** Othman Zuhir, Muzaffar Apipi, Jaswinder Singh, Yee Chin Lim

**Affiliations:** Department of Oral and Maxillofacial Surgery, Selayang Hospital (Ministry of Health), Batu Caves, Selangor, Malaysia

**Keywords:** case report, cellulitis, emphysema, intermaxillary fixation, mandible fracture

## Abstract

Facial cellulitis with palpable crepitus is a rare complication following a nondisplaced angle of mandible fracture. We report a case of a male in his mid-20s who presented with suspected emphysematous cellulitis of the face 3 days after an assault. Clinical examination revealed diffuse facial swelling with palpable subcutaneous crepitus over the left cheek and submandibular region. The patient had trismus and suppuration of the lower left third molar but no intraoral soft tissue injury. An orthopantomogram (OPG) showed a left nondisplaced angle of mandible fracture and multilocular bubble-like radiolucencies, suggesting submasseteric and submandibular gas accumulation. Due to financial constraints, advanced imaging and histological evaluation were not performed, limiting diagnostic certainty. Incision and drainage were performed, followed by intermaxillary fixation (IMF), and the infection was resolved with intravenous antibiotics. Open reduction internal fixation (ORIF) was not pursued due to cost limitations, but the fracture healed successfully within 6 weeks of IMF. This case underscores the importance of recognising gas-forming infections following mandibular trauma, the diagnostic challenges in resource-limited settings, and the role of early intervention in preventing severe complications.

## 1. Introduction

Mandibular fracture is one of the most frequently encountered injuries in the maxillofacial region [1]. While typically manageable, infection is a potential complication associated with mandibular fractures, which can worsen patient outcomes [1]. Several factors contribute to an increased risk of infection, including delayed presentation for treatment, poor oral hygiene at the time of injury, and inadequate initial management strategies [2].

A rare yet serious complication following mandibular fractures is subcutaneous facial emphysema, characterised by trapped gas within the facial soft tissues, with an incidence rate between 0.43% and 2.34% [3, 4]. Even rarer is facial cellulitis in the presence of trapped gas, which can mimic necrotising fasciitis—an aggressive, life-threatening infection of the fascia and subcutaneous tissue requiring prompt intervention [5]. Facial cellulitis and subcutaneous emphysema coexistence can present significant diagnostic challenges, as differentiating between these conditions is essential for guiding management. The condition of emphysematous cellulitis, although described in other anatomical sites [6, 7], remains underreported in the facial region.

This case report describes an unusual presentation of possible emphysematous cellulitis of the face following a nondisplaced mandibular angle fracture. It highlights the importance of considering this rare complication in the differential diagnosis of facial swelling after a mandibular trauma. We discuss the challenges encountered in diagnosing and managing this case, particularly in resource-limited settings.

## 2. Case Presentation

An otherwise fit and healthy man in his mid-20s presented to the emergency department with left facial swelling. He had allegedly been assaulted with a fist on his face during a robbery attempt in the late evening 3 days prior. He denied any loss of consciousness, vomiting, or nausea. In the morning following the incident, he developed mild swelling on the left side of his face. He attended a private general practitioner (GP) surgery and was given tranexamic acid and nonsteroidal anti-inflammatory drugs (NSAIDs). The next day, he developed odynophagia, which led to reduced oral intake. He consulted a different GP, who then prescribed him cefuroxime and dexamethasone. On the third day, the patient developed a trismus, prompting an emergency department visit.

### 2.1. Clinical Findings

**Figure 1:**
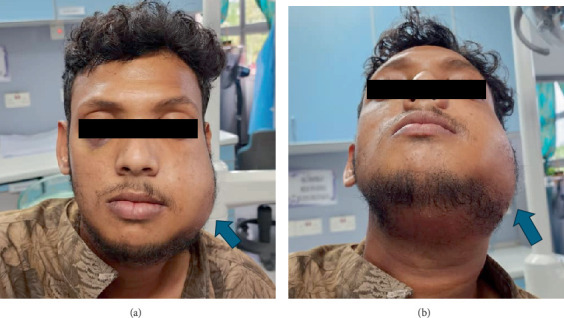
(a) Front view of the diffused swelling on the left side of the face (blue arrow). (b) Worm's eye view of the patient indicates the left submandibular and submasseteric swelling (blue arrow).

During the initial assessment, the patient was afebrile with unremarkable vital signs. Facial asymmetry was evident with a diffused, warm, tender, and fluctuant swelling (8 cm × 6 cm) on the left side. The swelling involved the left zygomatic arch superiorly, submandibular region inferiorly, lip commissure anteriorly, and preauricular area posteriorly (Figure 1). Subcutaneous crepitus was present on palpation. Palpating any step deformity of the left mandible was challenging due to the swelling. The patient also reported paraesthesia in the left mental region. The mouth opening was measured at 22 mm. There were signs of dysphagia but no chest pain, dysphonia, or dyspnoea, suggesting no airway involvement.

Intraoral examination revealed no occlusal derangement or step deformity, but the left retromolar trigone was tender. Haematoma was visible on the left sublingual region. The patient had buccal swelling with crepitus and thick yellowish purulent discharge on palpating the lower left third molar gingivae. The discharge increased upon milking of the submandibular swelling.

### 2.2. Diagnostic Assessment

**Figure 2:**
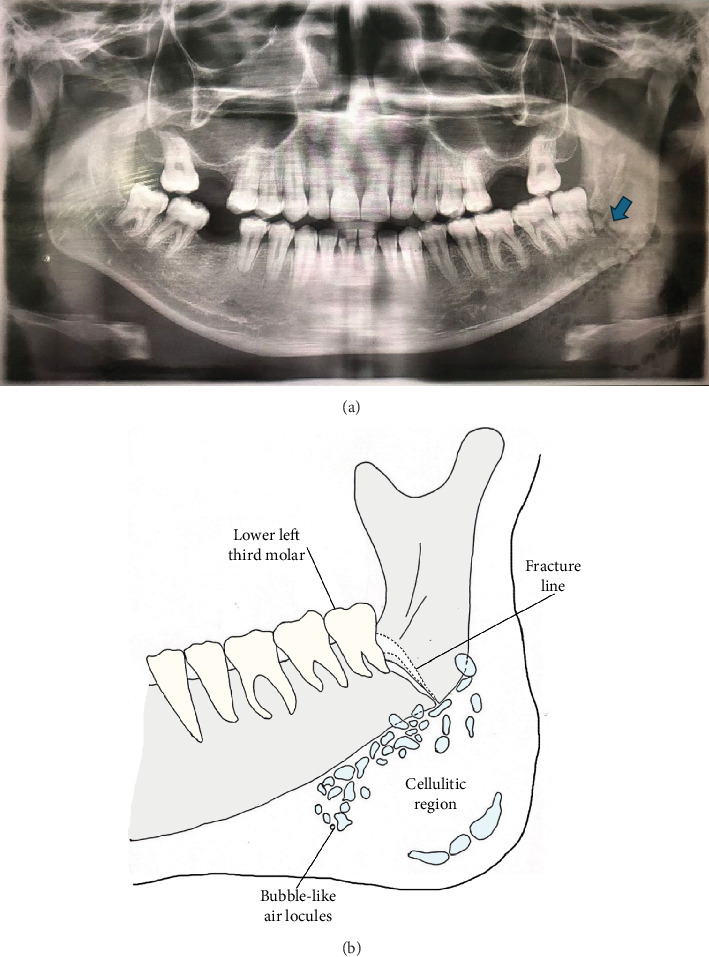
(a) The OPG shows a favourable nondisplaced fracture of the left angle of the mandible (blue arrow), extending from the inferior border of the mandible to the socket of the lower left third molar. (b) The drawn diagram illustrates the multiple bubble-like air locules in the cellulitic region.

An orthopantomogram (OPG) revealed a favourable nondisplaced fracture of the left angle of the mandible involving the distally impacted lower left third molar (Figure 2). Importantly, multilocular bubble-like radiolucencies were also present, confirming the presence of gas within the soft tissues. Although a CT scan could have provided more detailed information regarding the pus collection, the patient declined this due to financial constraints.

Blood investigations, including full blood count (FBC), urea and electrolytes (U&Es), and C-reactive protein (CRP), showed signs of leucocytosis, hypernatraemia, and increased inflammatory markers. A laboratory risk indicator for necrotising fasciitis (LRINEC) score to differentiate between severe cellulitis and necrotising fasciitis yielded a score of 7 (Table 1) [5]. This score, indicating an intermediate risk of developing necrotising fasciitis, highlighted the importance of close clinical monitoring with a low threshold for surgical intervention if signs of worsening infection develop [5].

The pus sample sent for microscopy, culture, and sensitivity (MC&S) testing revealed inconclusive mixed growth of gram-positive cocci and gram-negative rods. While it did not identify a specific causative organism, it supported the need for broad-spectrum antibiotic therapy to cover potential pathogens while awaiting the patient's clinical response to treatment.

Overall, the combination of clinical findings, positive gas presence on imaging, and elevated inflammatory markers led to the working diagnosis of possible emphysematous cellulitis of the face secondary to an untreated nondisplaced left angle of the mandible fracture.

### 2.3. Therapeutic Intervention

We took a multifaceted approach to address the mandibular fracture and emphysematous facial cellulitis.

The patient was presented with two options for treating the mandibular fracture: open reduction internal fixation (ORIF) or 6 weeks of intermaxillary fixation (IMF) via closed reduction. Due to financial limitations, he chose the latter. Thus, the Erich arch bars were affixed on the upper and lower arches and stabilised with double-loop interdental wiring. His lower left third molar, showing signs of pericoronitis, was extracted before IMF placement to prevent it from serving as a potential source of infection [8]. The removal outweighed the option of retaining the lower left third molar to obtain stability of the fracture segments [9].

Immediate management of the cellulitis involved intraoral incision and drainage (I&D) to drain the abscess accumulated in the buccal sulcus. However, it yielded minimal pus. Extraoral I&D followed, draining foul-smelling, brown-coloured pus. Corrugated rubber drains were placed to ensure ongoing drainage, and the site was irrigated with povidone and gentamycin twice daily. Considering local guidelines and the broad spectrum of potential pathogens, intravenous (IV) cefuroxime was initiated. We also prescribed analgesics and NSAIDs for pain and inflammation.

**Figure 3:**
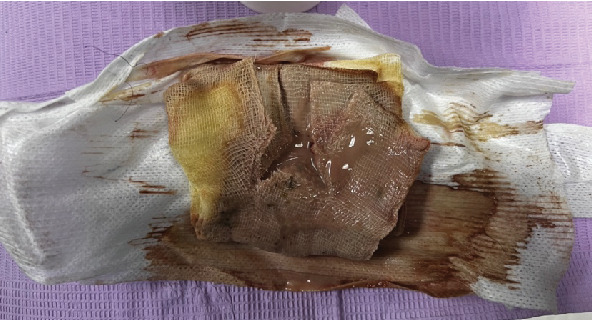
Brown pus collected on the dressing on the seventh day.

However, after several days, the patient's condition remained unimproved, and the inconclusive MC&S result suggested potential antibiotic resistance. Furthermore, a moderate amount of brown-coloured pus was collected on the seventh day (Figure 3), as the intraoral and extraoral sites communicated, leading to possible reinfection at the fracture site. A second debridement was, thus, performed on the reinfected sites to remove necrotic tissue and pus. We switched the antibiotic regimen to clindamycin for its broader coverage against anaerobes and gram-positive cocci commonly found in submandibular space infections. These measures improved the patient's condition. We removed the drains on the eighth day and loosely sutured the extraoral site on the ninth day.

After 10 days on IV antibiotics, the regimen was transitioned to oral antibiotics before hospital discharge. The patient was advised against smoking, contact sports, and Valsalva manoeuvres.

### 2.4. Follow-Up and Outcomes

During the 4-week follow-up appointment, the patient was in no pain, and facial swelling had resolved. There were no signs of facial asymmetry or step deformity. The I&D sites healed well, with minimal scarring.

**Figure 4:**
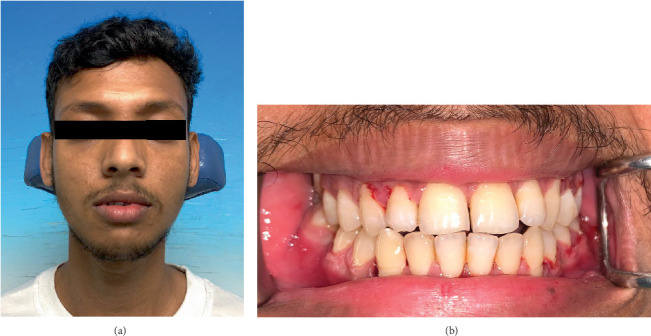
(a) Front view of the patient on the 49th day, indicating facial symmetry with resolved swelling. (b) His occlusion was at maximum intercuspation following 6 weeks of IMF.

After 6 weeks, the IMF was removed. Upon removal of the Erich arch bars and interdental wires, his mouth opening was 45 mm, and his occlusion was at maximum intercuspation (Figure 4). He was advised on oral hygiene, provided with a jaw exercise routine, and instructed to avoid contact sports for another 2 months.

## 3. Discussion

This case of suspected emphysematous cellulitis of the face following a mandibular fracture in a young patient underscores the diagnostic challenges and therapeutic dilemmas encountered in resource-limited settings. It also highlights the rarity of this condition in the facial region, particularly when associated with trauma.

### 3.1. Diagnostic Uncertainty

The diagnostic uncertainty in this case was primarily due to the lack of advanced imaging modalities, such as CT or MRI, which are critical for the three-dimensional localisation of gas and delineation of soft tissue involvement. Without these tools, the precise aetiology of the gas and infection remains speculative. It is plausible that air bubbles introduced during trauma persisted and created an environment conducive to bacterial growth, leading to infection. Trapped gas within the facial soft tissues has been reported in several cases following dental procedures and maxillofacial injuries [3, 10, 11]. Air influx into the subcutaneous layer can occur via high-speed dental handpieces or Valsalva manoeuvres, potentially spreading into fascial planes. Alternatively, the gas formation could have been secondary to anaerobic bacterial activity, as seen in necrotising fasciitis or gas gangrene, which share overlapping clinical features of rapid tissue destruction and subcutaneous gas accumulation [12].

### 3.2. Necrotising Fasciitis, Gas Gangrene, and Suspected Emphysematous Cellulitis

The clinical presentation in this case necessitated careful consideration of necrotising fasciitis and suspected emphysematous cellulitis. Necrotising fasciitis is typically characterised by polymicrobial or monomicrobial infections with systemic toxicity [13], whereas gas gangrene, often caused by *Clostridium* species, is associated with muscle necrosis and hallmark features such as haemorrhagic bullae and profound systemic toxicity [14]. The patient's intermediate LRINEC score suggested a possible risk of necrotising fasciitis. However, the absence of key diagnostic features such as skin discolouration and bullae made this diagnosis unlikely [13]. Similarly, the lack of hallmark systemic signs of gas gangrene, such as haemorrhagic bullae or severe systemic toxicity, combined with the patient's rapid clinical improvement on antibiotics, reduced the likelihood of gas gangrene [14]. Given the rarity of emphysematous cellulitis in the facial region, differentiating it from necrotising infections was crucial. Identifying gas-producing bacteria such as group *A streptococcus*, *Clostridium* species, MRSA, or anaerobic species like *Prevotella* and *Porphyromonas* would have been valuable in confirming the diagnosis [3, 15, 16]. However, the culture results were inconclusive, and histological analysis of debrided tissue was not performed due to resource constraints. These limitations necessitated a working diagnosis of emphysematous cellulitis based on clinical and radiographic findings.

### 3.3. Antibiotic Management

The initial empirical use of cefuroxime was ineffective, likely due to beta-lactam resistance. The subsequent switch to clindamycin proved effective, underscoring the importance of targeting aerobic and anaerobic pathogens in suspected gas-producing infections. This case highlights the critical role of early broad-spectrum antibiotic therapy, particularly when culture results are delayed or inconclusive. Delayed presentation beyond the optimal 72-h prophylactic window further complicated the management and may have contributed to infection progression [17].

### 3.4. Impact of Financial Constraints

This case also raises broader issues regarding the impact of financial constraints on patient care. Advanced imaging studies, which could have provided detailed insights into gas distribution and tissue involvement, were not performed due to the patient's inability to afford them. The reliance on OPG imaging, while helpful in identifying the fracture and gas presence, was inadequate for comprehensive surgical planning. Similarly, the decision to opt for closed reduction instead of ORIF was dictated by financial considerations despite the latter being the gold standard for managing mandibular fractures. Addressing these challenges requires the development of cost-effective diagnostic protocols and improved access to financial support programmes to ensure equitable care.

## 4. Conclusion

This case report highlights the rare presentation of possible emphysematous cellulitis of the face following a nondisplaced mandibular fracture and the associated challenges of managing such cases in resource-limited settings. The absence of advanced imaging and histopathological evaluation limited the diagnostic certainty, while financial constraints influenced diagnostic and therapeutic pathways. This case underscores the importance of maintaining a high index of suspicion for gas-producing infections following mandibular trauma and the necessity for early intervention with broad-spectrum antibiotics and surgical debridement. Clinicians managing similar cases in resource-limited settings must rely heavily on clinical judgment while advocating for increased accessibility to diagnostic and therapeutic resources. Moving forward, early recognition, precise differentiation between potential conditions, and judicious antibiotic selection remain paramount in optimising outcomes for such complex presentations. Additionally, this case highlights the need for cost-effective diagnostic strategies and expanded financial support mechanisms to bridge gaps in care, ultimately improving patient outcomes in low-resource settings.

## Figures and Tables

**Table 1:** The parameters used to calculate the patient's LRINEC score [5]. A total LRINEC score of less than 5 is classed as a low risk of developing necrotising fasciitis, whereas a score of 6–7 has an intermediate risk, and a score of 8 or greater is a high risk. In this case, the patient scored 7, indicating an intermediate risk of developing necrotising fasciitis.

**Variable (unit)**		**Score**	**Patient's score**
C-reactive protein (mg/L)	< 150	0	4
	≥ 150	4	

Total leucocyte count (thousands/mm^3^)	< 15	0	1
	15–25	1	
	> 25	2	

Haemoglobin (g/dL)	> 13.5	0	0
	11–13.5	1	
	< 13.5	2	

Serum sodium (mmol/L)	≥ 135	0	2
	< 135	2	

Serum creatinine (*μ* mol/L)	≤ 141	0	0
	> 141	2	

Glucose (mmol/L)	≤ 10	0	0
	> 10	1	

Total LRINEC score			7

## Data Availability

Information and data in this case report are available from the corresponding author upon reasonable request. They are not publicly available due to ethical issues.
